# PROGRESSION TO FRAILTY – THE ROLE OF MUSCLE RESILIENCE

**DOI:** 10.1093/geroni/igae098.4057

**Published:** 2024-12-31

**Authors:** LaDora Thompson, Lais Perazza

**Affiliations:** Boston University, Boston, Massachusetts, United States; Boston University, Boston University, Massachusetts, United States

## Abstract

The impact of a stressful event within the progression to frailty is unknown. In this study we determined the response to an acute stress in Robust, Prefrail, and Frail mice. We hypothesized the Robust mice would show greater resilience compared to Prefrail and Frail mice following a stressful event; whereas the Prefrail mice would show an intermediary resilience status. 17-27-month-old C57BL/6 female mice were classified as Robust, Prefrail or Frail using the Vitality Phenotype (strength, endurance, activity, walking speed, health deficits.) Stress was induced by downhill running (DR). Resilience was determined by muscle contractility and bone mineral density (BMD). 38% of cohort was classified as Prefrail, 33% as Frail and 29% as Robust. Performance parameters were different between the 3 categories of frailty. Tetanic force (soleus) in Frail mice was significantly reduced compared to Prefrail and Robust mice. When tetanic force was normalized to muscle mass, the frailty-driven reduction in force was still observed, suggesting impairment in protein quality. BMD was similar between Prefrail and Frail, while both differed from Robust, suggesting that BMD loss occurred early in the progression to frailty. Following DR, the Robust and Prefrail mice showed a significant reduction in tetanic force compared to non-damaged control Robust and Prefrail mice. In contrast, soleus tetanic force in Frail mice following DR was not different between non-damaged control Frail mice, likely due to the already preexisting muscle dysfunction. In sum, our data suggests that resilience after acute stress is dependent on the frailty status.

